# Oxytocin and Vasopressin Involved in Restraint Water-Immersion Stress Mediated by Oxytocin Receptor and Vasopressin 1b Receptor in Rat Brain

**DOI:** 10.1371/journal.pone.0023362

**Published:** 2011-08-17

**Authors:** Dong-Qin Zhao, Hong-Bin Ai

**Affiliations:** Key Laboratory of Animal Resistance of Shandong Province, College of Life Sciences, Shandong Normal University, Shandong Province, People's Republic of China; National Institutes of Health, United States of America

## Abstract

**Aims:**

Vasopressin (AVP) and oxytocin (OT) are considered to be related to gastric functions and the regulation of stress response. The present study was to study the role of vasopressinergic and oxytocinergic neurons during the restraint water-immersion stress.

**Methods:**

Ten male Wistar rats were divided into two groups, control and RWIS for 1h. The brain sections were treated with a dual immunohistochemistry of Fos and oxytocin (OT) or vasopressin (AVP) or OT receptor or AVP 1b receptor (V_1b_R).

**Results:**

(1) Fos-immunoreactive (Fos-IR) neurons dramatically increased in the hypothalamic paraventricular nucleus (PVN), the supraoptic nucleus (SON), the neucleus of solitary tract (NTS) and motor nucleus of the vagus (DMV) in the RWIS rats; (2) OT-immunoreactive (OT-IR) neurons were mainly observed in the medial magnocellular part of the PVN and the dorsal portion of the SON, while AVP-immunoreactive (AVP-IR) neurons mainly distributed in the magnocellular part of the PVN and the ventral portion of the SON. In the RWIS rats, Fos-IR neurons were indentified in 31% of OT-IR neurons and 40% of AVP-IR neurons in the PVN, while in the SON it represented 28%, 53% respectively; (3) V_1b_R-IR and OTR-IR neurons occupied all portions of the NTS and DMV. In the RWIS rats, more than 10% of OTR-IR and V_1b_R-IR neurons were activated in the DMV, while lower ratio in the NTS.

**Conclusion:**

RWIS activates both oxytocinergic and vasopressinergic neurons in the PVN and SON, which may project to the NTS or DMV mediating the activity of the neurons by OTR and V_1b_R.

## Introduction

Restraint water-immersion stress (RWIS) is considered to be a mixture of physical and psychological stressor, and this stimulation in conscious rats induces behavioral responses (anxiety, scrabble, outrage and cry), hypothermia and vagally-mediated gastric hypercontractility, gastric acid hypersecretion and gastric mucosal lesions within a few hours [1]–[2]. We previously used the model in rats to study the neuronal pathways activated during gastric dysfunction mechanical stimulation. After different durations of RWIS, neuronal activation, demonstrated by Fos-immunoreactivity (Fos-IR), was found significantly increased in specific brain areas, such as the medullary visceral zone [dorsal motor nucleus of the vagus (DMV), nucleus of solitary tract (NTS), area postrema (AP) and nucleus ambiguous (NA)] and the hypothalamus [paraventricular nucleus (PVN) and supraoptic nucleus (SON)] [3]–[4]. These results suggest the neuronal hyperactivity of the NTS, DMV and AP may be one of the primary central mechanisms of the gastric dysfunctions induced by the RWIS, while the neuronal hyperactivity of PVN and SON may be one of the higher central mechanisms. Brainstem circuits regulating gastric function have been studied widely [5]–[7], but little is known about the higher central neuronal mechanisms of the gastric dysfunction induced by the RWIS. Previous studies indicate that the abnormalities of gastric functions induced by RWIS are not due to the hyperactivity of the hypothalamo-pituitary-adrenal (HPA) axis and the sympathetic adrenamedullary system, but due to the hyperactivity of vagal parasympathetic efferents, which largely originating in the DMV and partly in the NA [3], [5]–[9]. It seems that the hyperactivity of the DMV, NTS and AP leads to gastric disfunction. But, our previous researches demonstrated that electrical and chemical stimulations of the DMV and NA inhibited gastric motility [10]–[11]. So, whether the hyperactivity of the higher centre of the anterior hypothalamus relieves the inhibition of gastric motility mediated by the primary nerve centre during the RWIS? If so, what are the neurotransmitters released by the anterior hypothalamus neurons?

The PVN and SON are the main nuclei of the anterior hypothalamus, which might be stimulus-dependent [8], [12]. While the SON is composed exclusively of magnocellular neurons, the PVN is more heterogeneous and includes also parvocellular neurons [13]. The parvocellular region of the PVN, based on the cell density and cell size, can be divided into the anterior (PaAP), medial (PaMP), posterior (PaPo) and periventricular (Pe) subdivisions. The magnocellular region of the PVN is characterized by the compact clustering of the large cells and can be divided into lateral (PaLM) and medial (PaMM) subdivisions [14]. Vasopressin (AVP) and oxytocin (OT) are two structurally related nonapeptides synthesized mainly in the magnocellular neurons of the PVN and SON [15]–[17], and may act as neurotransmitters and/or neuromodulators which are considered to be related to gastric functions [18] and the regulation of stress response [19]–[27]. One aim of the present study was to find out whether the activated neurons in the PVN and SON of rats induced by RWIS were OT and AVP neurons.

Furthermore, to illustrate the OT and AVP neurons in the anterior hypothalamus taking part in the mediation of signals induced by RWIS, the other aim of this study was to determine whether the phenotypic nature of activated neurons in the medullary visceral zone were AVP sensitive or OT sensitive neurons, where AVP or OT receptors located. To date, three types of AVP receptors have been described: V_1a_,V_1b_ and V_2_ receptors, whereas OT receptors only one [28]–[30]. Receptors for OT and AVP have been found in various regions of the rat brain, including the hypothalamic nuclei, NTS, DMV and so on [29]–[32]. In the central nervous system, the action of AVP seems to be predominantly mediated by V_1_-type AVP receptors [33]–[34].

So, in the present study, the extent of activation as well as the distribution of the activated neurons, mainly in PVN, SON, DMV and NTS, was determined by immunohistochemistry employing an antiserum specific for Fos protein, which is known as a marker of neural activation [35]. To evaluate the role of OT and AVP during the RWIS, the phenotype nature of activated neurons was determined by a double immunohistochemical method for co-locations of Fos with either AVP, OT, V_1b_R or OTR specific antibodies.

## Materials and Methods

### Preparation of animals

Male Wistar rats (Experimental Animal Center of Shandong University, Jinan, China), weighing 180–200 g, were housed two per cage at an ambient temperature of 22±2°C under a normal day/night cycle with foot and water available ad libitum before initiation of the RWIS. Experiments were initiated at least 7 days after arrival. Before stress, the rats were fasted for 24 hours, but allowed free access to water.

### Stress protocols

Ten rats were randomly divided into two groups in accordance with the duration of RWIS, respectively: the RWIS group and the control group. RWIS was performed as previously described [36]. Briefly, under light ether anesthesia, the four limbs of each rat in the stressed group were bounded gently but securely on a wooden board by use of medical adhesive tape. When the rats were conscious, they were vertically immersed in water (21±1°C) to the level of the xiphoid for 1 h. Unstressed rats, as a control group, were not stressed but were otherwise under identical conditions. To avoid the effect of diurnal variations on the Fos expression, the experiment was performed between 9:00 and 11:00 a.m. All procedures were performed in accordance with the ethic guidelines of the International Association for the Study of Pain [37].

### Tissue processing

At the end of the procedure, the rats were deeply anesthetized by intraperitoneal injections of over sodium pentobarbital (100 mg/kg body weight) and perfused via the ascending aorta with 200 ml 0.01 mol/L phosphate buffered saline (PBS, pH 7.4) followed by 500 ml 4% paraformaldehyde, 0.1% glutaraldehyde and 14% saturated picric acid in 0.1 mol/L phosphate buffer (pH 7.4). After perfusion, the brain was removed and post-fixed at 4°C for 4 h in the same fixative, and then infiltrate with 20% sucrose in 0.1 mol/L phosphate buffer for 48 h at 4°C. Series of frozen coronal sections of the hypothalamus and medullary visceral zone were cut at 30 µm in a cryostat and collected into 0.01 mol/L PBS.

### Immunohistochemistry

The immunoreaction of Fos plus neuropeptide, AVP and OT, and their receptor, V1b and OTR, was detected by a dual SP (streptavidin-biotin-peroxidase) immunohistochemical technique. Briefly: (1) Free floating sections were rinsed in 0.01 mol/L PBS followed by a preincubation in methanolic 3% H_2_O_2_ for 30 min at room temperature to eliminate endogenous peroxidase activity. (2) After rinsing in 0.01 mol/L PBS, the sections were incubated with blocking buffer, containing 5% normal goat serum and 0.3% Triton X-100 in 0.01 mol/LPBS for 30 min, and then were incubated with rabbit anti-c-Fos antibody (sc-52, Santa Cruz Biotechnology Inc, USA), diluted 1∶2000 in 0.01 mol/L PBS containing 3% normal goat serum (NGS) and 0.3% TritonX-100 for 24 hours at 4°C. (3) At the end of this incubation period, rinsing in 0.01 mol/L PBS, the sections were incubated with the biotinylated goat anti-rabbit IgG (Zymed Laboratories Inc, USA) for 1 h at room temperature and next PBS rinse were followed by incubation with streptavidin-biotin-horseradish peroxidase complex (Zymed Laboratories Inc, USA) for 1 h at room temperature. (4) After several rinsing in PBS, the sections were submitted to a diaminobenzidine hydrochloride (DAB, Sigma Chemical Co. St Louis, MO, USA), intensified with 0.05% cobalt chloride and 0.05% nickel ammonium sulfate for 4–5 min. This method produces a blue-black nuclear reaction product. (5) The Fos-immunoreactive (Fos-IR) sections were rinsed and incubated for 24 h with rabbit anti-AVP (1∶2000, Abcam plc 332 Cambridge Science Park, Cambridge, CB4 0WN, UK), mouse anti-OT (1∶2000, Abcam plc 330 Cambridge Science Park, Cambridge, CB4 0FL, UK), rabbit anti-V_1b_R (1∶200, International Laboratory USA) or anti-OTR (1∶200, USCNLIFE Science CO, USA) in 3% NGS and 0.3% TritonX-100 for 48 hours at 4°C. (6) and (7) were the same as (3) and (4). (8) The visualization of the immunoreactive products was obtained by reaction with unintensified DAB that produces a brown reaction product. (9) Lastly, the free-floating sections were mounted on gelatin-coated glass slides, air-dried overnight, dehydrated in a series of alcohols, cleared in xylene and placed under a coverslip with Permount.

### Evaluation of immunostaining

Pictures of brain sections were taken under identical conditions with a BX51 Olympus microscope (Olympus Corporation, Japan) coupled to an Olympus DP70 camera. The nomenclature and nuclear boundaries defined in the rat brain stereotaxic atlas of Paxinos & Watson [38] were used in this study. For quantitative assessment, the number of immunoreactive neurons using Image-Pro Plus 6.0 (Media Cybernetics Inc, USA), was counted at three levels. For the PVN: the anterior portion (Bregma, -1.08 to -1.32 mm), the medial portion (−1.72 to −1.92 mm), and the posterior portion (Bregma, −2.04 to −2.16 mm). For the NTS and DMV: rostral (Bregma, −12.96 to −13.32 mm), intermediate (Bregma, −13.80 to −14.04 mm), and caudal (Bregma, −14.52 to −14.76 mm) [7]. The immunoreactive neurons in SON were counted at one level (Bregma, −0.92 to −1.44 mm). In each nucleus, 3 kinds of neurons were counted, which were Fos-IR nuclei, AVP or OT or V_1b_R or OTR-IR neurons and Fos+ AVP or OT or V_1b_R or OTR -IR neurons.

The number of immunoreactive neurons was counted in three near sections per animal and the average values of them in 0.01 mm^2^ are reported as the number of immunoreactivity.

### Statistical analysis

Counting was performed in five rats for each condition and the data obtained from each animal were used to calculate group means ±SEM. The statistical procedures were performed with SPSS13.0 software (SPSS, Chicago, IL, USA). Statistical analysis about data in different portions of the PVN, NTS and DMV were performed by two-way analysis of variance (ANOVA) followed by S-N-K's *post hoc* test individually. Statistical analysis about data in the SON was performed by Student's *t*-test. *P*<0.05 and *P*<0.01 were considered statistically significant.

## Results

### RWIS induced Fos expression in specific brain nuclei

Changes in neural activity were assessed by monitoring Fos expression in the hypothalamus and the medullary visceral zone after RWIS for 1 h. The response to RWIS and the pattern of distribution of Fos-IR were essentially the same as previously reported by us, while few Fos-IR neurons appeared in the same area in unstressed rats [3]. Briefly, the activated neurons mainly occupied the anterior hypothalamus, including the PVN and SON, and the medullary visceral zone, including the NTS and DMV. SO, in the present study, we focused on the hypothalamic areas containing large populations of vasopressinergic, oxytocinergic neurons responsive to RWIS and medullarry areas containing the AVP or OT sensitive neurons responsive to RWIS.

### Distribution of Fos and Fos+OT and Fos+AVP immunoreactive neurons in the hypothalamus

#### PVN

**Figure 1:**
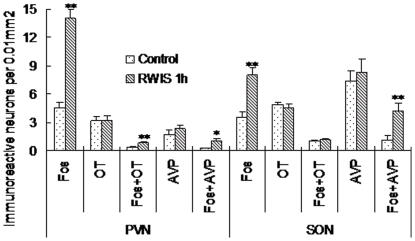
Cells count (cells per 0.01 mm^2^) of Fos-IR and OT-IR or AVP-IR neurons in the PVN and SON. n = 5 rats per group. Each group represents mean±SEM. ** *P*<0.01, * *P*<0.05 *vs* the control group.

**Figure 2:**
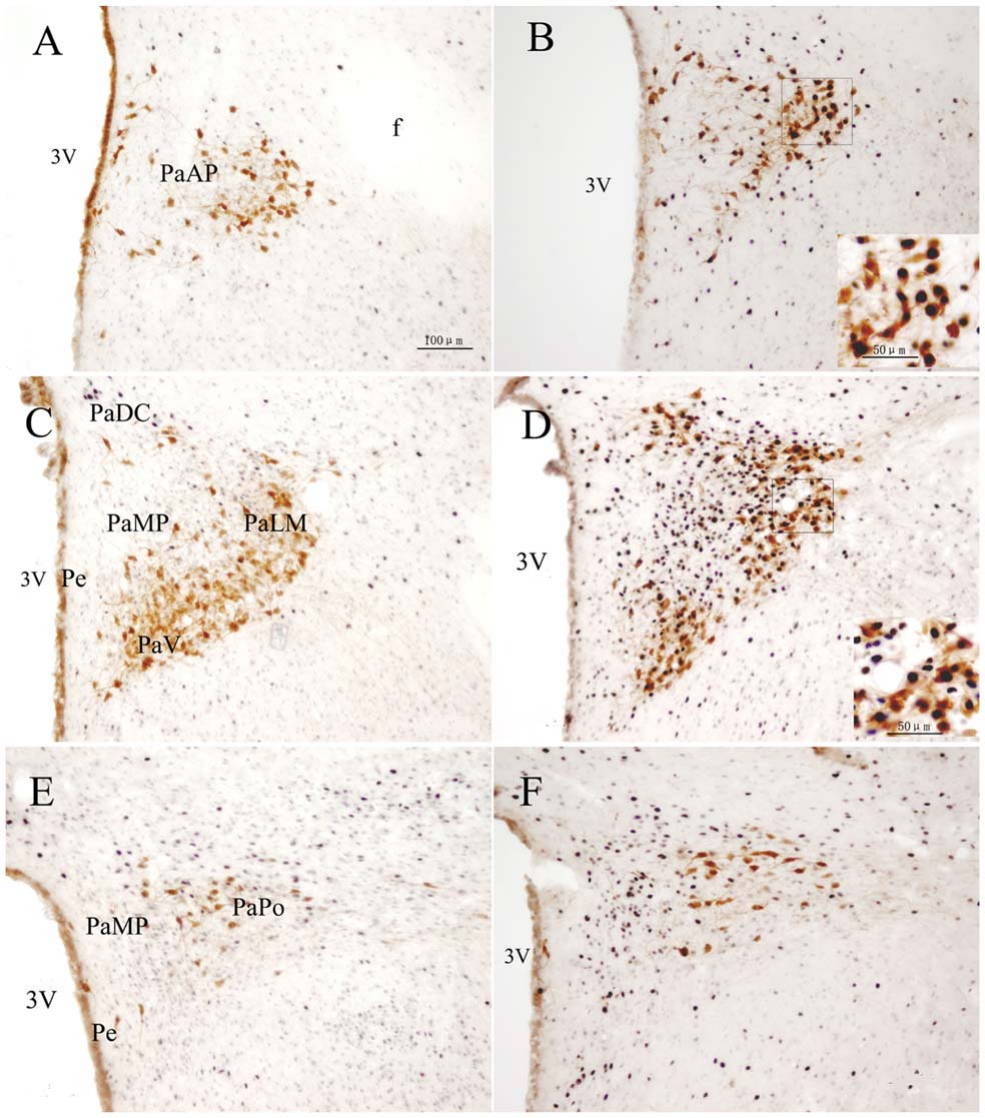
Double immunohistochemical staining of Fos-IR and OT-IR neurons in the PVN. A, B: the anterior. C, D: the medial. E, F: the posterior. A, C, E: the unstressed rats. B, D, F: rats induced by restraint water-immersion stress for 1 h. The inserts show a higher magnification (400X) of cells in the small boxes. PaAP: anterior parvicellular part of the PVN; PaLM: lateral magnocellular part of the PVN; PaV: the ventral part of the PVN; PaMP: medial parvocellular part of the PVN; PaDC: dorsal cap of the PVN; Pe: periventricular of the PVN; 3V: 3rd ventricle. Bar in all panels is 100 µm, while in the inserts is 50 µm.

RWIS for 1 h increased Fos-IR nuclei to 7.9±1.3 (cells per 0.01 mm^2^) in the PVN compared with 3.3±0.3 in unstressed rats, *i.e.* 2.4-fold (*P*<0.01) (Figure 1). Maximal number of Fos-IR neuclei were observed in the medial portion in the PVN, while comparably less of them was present in its anterior and posterior portions, the difference was significant (*F*_2,12_ = 53.913, *P* = 0.000). Numerous Fos-IR nuclei occurred explicitly in the PaMP, PaMM and PaLM. Fos-IR nuclei in the PaMP were heterogeneous in size and had irregular profiles, while those in the PaLM were large round and more homogeneous in size. Besides these locations, some Fos-IR neurons were also found in the lateral portion of the PaAP, the dorsal cap (PaDC), ventral (PaV) subdivisions of the PVN. Similarly, a few of Fos-IR neurons were observed in the Pe and PaPo (Figure 2).

OT-IR neurons within the PVN were mostly located in the medial portion, including the PaMM, where they aggregated into a compact cell cluster, and the PaLM, where they had fusiform or round profiles with number of neuronal processes (Figure 2). Besides these locations, the lateral portion of the PaAP, the PaDC and PaV had a moderate number of OT-IR neurons and a few were scattered in the PaMP, PaPo and Pe, where closed to the wall of the third ventricle (Figure 2). Fos+OT-IR neurons in the PVN were mainly located in the PaMM, PaLM and the PaV, and to a less extent, in the lateral portion of the PaAP, PaDC and PaMP. In the PaPo almost no double staining cells were found. Overall, in the PVN, Fos+OT-IR neurons represented in 31.40%±0.76% of total OT-IR neurons in the RWIS group, and had significant difference compared with 11.74%±3.71% in the control group (*P*<0.01).

**Figure 3:**
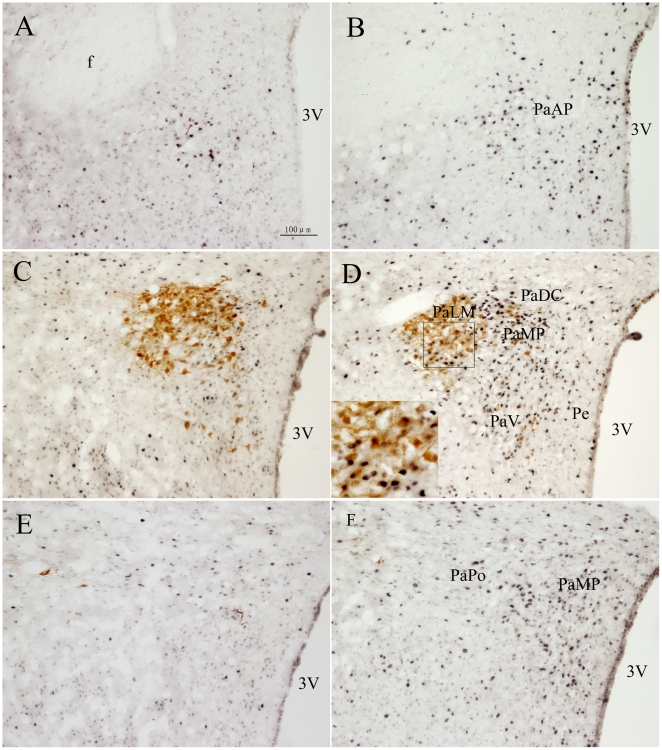
Double immunohistochemical staining of Fos-IR and AVP-IR neurons in the PVN. A, B: the anterior. C, D: the medial. E, F: the posterior. A, C, E: the unstressed rats. B, D, F: rats induced by restraint water-immersion stress for 1 h. The inserts show a higher magnification (400X) of cells in the small boxes. The portions of the PVN was same as Figure2. 3V: 3rd ventricle. Bar in all panels is 100 µm, while in the inserts is 50 µm.

AVP-IR neurons mainly located in the magnocellular portion of the medial part of the PVN, including the PaMM and the PaLM, where most of Fos+AVP-IR neurons were observed (Figure 3). In the PaMP, PaDC as well as the PaPo, only scattered AVP-IR neurons were found, and almost no AVP-IR neurons located in the anterior portion of the PVN and the Pe (Figure 3). Fos+AVP-IR neurons in response to RWIS represented in 40.39%±6.78% of AVP-IR neurons while 14.21%±3.42% in the control group (*P*<0.01).

#### SON

**Figure 4:**
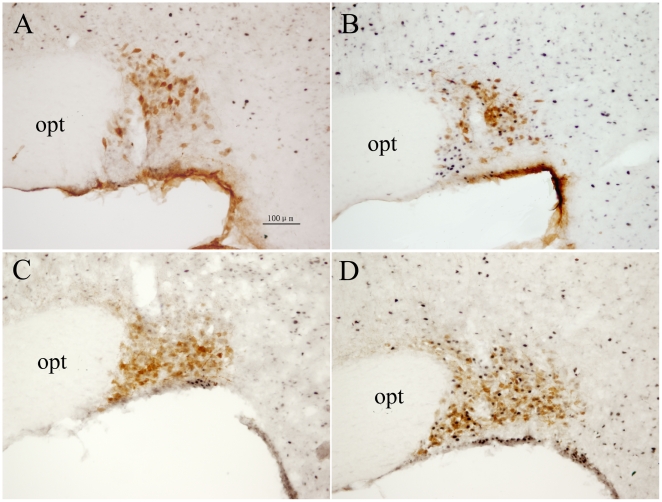
Double immunohistochemical staining of Fos-IR and OT-IR or AVP-IR neurons in the SON. A, C: the unstressed rats. A, B: OT-IR. C, D: AVP-IR. B, D: rats induced by restraint water-immersion stress for 1 h. OT-IR neurons were observed mainly in dorsal part of the SON, while AVP-IR neurons were mainly distributed in the ventral part of the SON. opt: optic tract. Bar in all panels is 100 µm.

Likewise in the case of Fos-IR nuclei in the PVN, the number of Fos-IR nuclei in the SON in the RWIS group (8.0±0.8 cells per 0.01 mm^2^) was significantly higher than that in the control group (3.5±0.6 cells per 0.01 mm^2^) (*P*<0.01) (Figure 1). In contrast to the PVN, Fos-IR nuclei in the SON were less abundant and evenly distributed. Morphometry showed that Fos-IR nuclei in the SON were large and round in similar size (Figure 4).

OT-IR neurons were observed mainly in dorsal part of the SON (Figure 4 A,B). Fos+OT-IR neurons in response to RWIS represented in 27.94%±3.05% of OT-IR neurons and 19.62%±2.88% in the control group (*P*<0.05).

Compared with the OT-IR neurons, AVP-IR neurons were mainly distributed in the ventral part of the SON (Figure 4 C,D). The percentage of Fos+AVP-IR in the total AVP-IR neurons in the RWIS rats was significantly increased by RWIS for 1 h (52.88%±4.53%) compared with that in the unstressed rats (19.44%±8.88%) (*P*<0.01).

### Distribution of Fos and Fos+OTR and Fos+V_1b_R immunoreactive neurons in the medullary visceral zone

#### DMV

**Figure 5:**
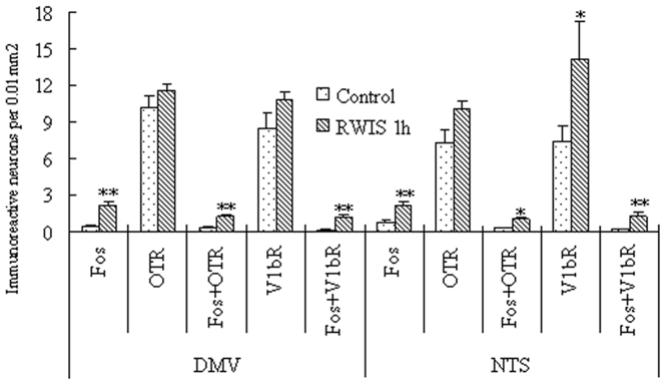
Cells count (cells per 0.01 mm^2^) of Fos-IR and OTR-IR or V_1b_R-IR neurons in the DMV and NTS. n = 5 rats per group. Each group represents mean±SEM. ** *P*<0.01, * *P*<0.05 *vs* the control group.

**Figure 6:**
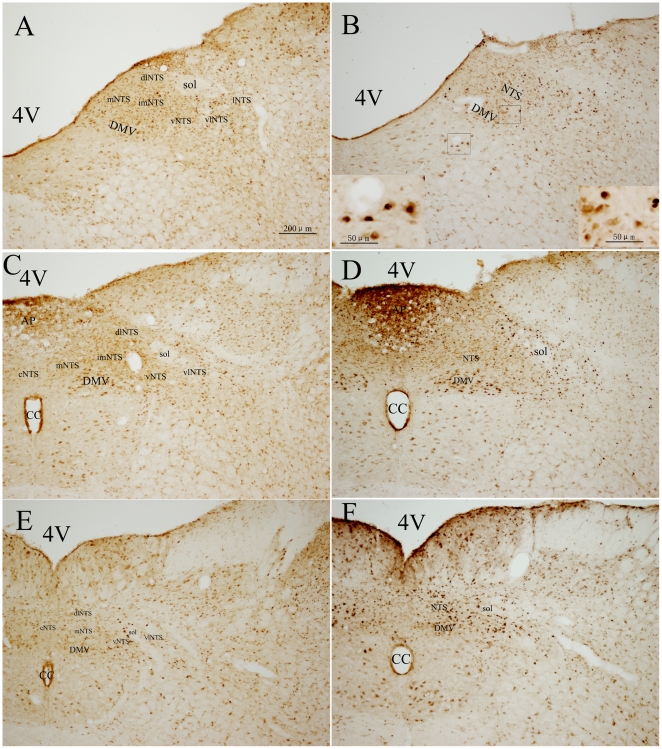
Double immunohistochemical staining of Fos-IR and OTR-IR neurons in the DMV and NTS. A, B: rostral. C, D: intermediate. E, F: caudal. A, C, E: Unstressed rats. B, D, F: rats induced by restraint water-immersion stress for 1 h. The inserts show a higher magnification (400X) of cells in the small boxes. cNTS: commissural nucleus of solitary tract; mNTS: medial nucleus of solitary tract; imNTS: intermediate nucleus of solitary tract; vNTS: ventral nucleus of solitary tract; vlNTS: ventrolateral nucleus of solitary tract; dlNTS: dorsolateral nucleus of solitary tract; 4V: 4rd ventricle; CC: central canal. Bar in all panels is 100 µm, while in the inserts is 50 µm.

**Figure 7:**
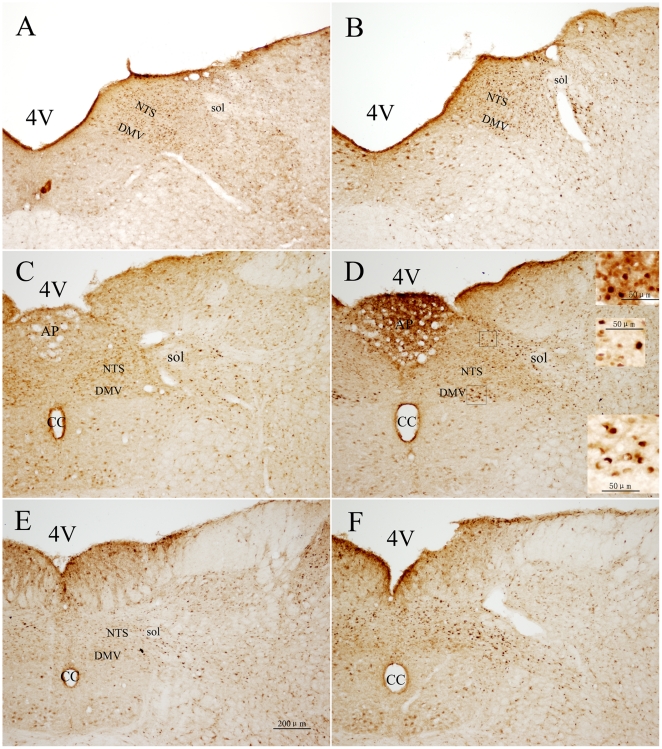
Double immunohistochemical staining of Fos-IR and V_1b_R-IR neurons in the the DMV and NTS. A, B: rostral. C, D: intermediate. E, F: caudal. A, C, E: Unstressed rats. B, D, F: rats induced by restraint water-immersion stress for 1 h. The inserts show a higher magnification (400X) of cells in the small boxes. The subdivision of the NTS was same as Figure 6. 4V: 4rd ventricle; CC: central canal. Bar in all panels is 100 µm, while in the inserts is 50 µm.

In the DMV, RWIS for 1 h induced a robust increase in Fos-IR nuclei by 4.2 times (2.1±0.5 *vs* 0.5±0.2 in the control group, cells per 0.01 mm^2^) (*P*<0.01) (Figure 5). The labeled neuronal nuclei in the DMV were large and round in similar size (Figure 6,7). The occurrence of Fos-IR nuclei was evident from the rostral to the caudal portions of the DMV. Maximal number of Fos-IR neuclei were found in the intermediate part of the DMV, while comparably less of them was present in its anterior and posterior portions, the difference was significant (*F*_2,12_ = 5.907, *P* = 0.015).

OTR-IR neurons were observed evidently from the rostral to the caudal portions of the DMV either in the RWIS rats or the unstressed rats (Figure 6). OTR-IR neurons in the DMV, evenly distributed, were large fusiform or round profiles with few number of neuronal processes. The major location of Fos+OTR-IR neurons was the intermediate part of the DMV, and less of them was present in its posterior and anterior portions, but the difference was not significant (*F*_2,12_ = 0.845, *P* = 0.454). Overall, in the DMV, Fos+OTR-IR neurons represented in 10.22%±0.54% of total OTR-IR neurons in the RWIS group, and compared with that in the control group (2.87%±0.34%), the difference was significant (*P*<0.01).

V_1b_R-IR neurons were also observed evidently from the rostral to the caudal portions of the DMV either in the RWIS rats or the unstressed rats (Figure 7). Compared with OTR-IR neurons, V_1b_R-IR neurons in the DMV were lesser in size and round profiles without any neuronal processes. The major location of Fos+V_1b_R-IR neurons was the intermediate part of the DMV, while less of them was present in its anterior and posterior portions, the difference was significant (*F*_2,12_ = 5.735, *P* = 0.018). Overall, in the DMV, Fos+V_1b_R-IR neurons represented in 10.72%±3.22% of total V_1b_R-IR neurons, compared with 2.87%±1.77% in the control group, the difference was significant (*P*<0.05).

#### NTS

In the NTS, there was an induction of Fos-IR nuclei in RWIS rats when compared with unstressed rats (2.1±0.1 *vs* 0.8±0.1 cells per 0.01 mm^2^, *P*<0.01) (Figure 5). The location of Fos-IR nuclei was evident from the rostral to the caudal portions of the NTS (Figure 6,7). There was no significant difference in the number of Fos-IR nuclei in any portion of the NTS, in spite of the occurrence of Fos-IR nuclei in the rostral and caudal portions being less numerous (*F* = 1.339, *P*>0.05). Fos-IR nuclei were mainly observed in the intermediate (imNTS) and ventrolateral (vlNTS) (Figure 6,7), along with a few stained cells were found in the ventral (vNTS) and the medial (mNTS) subnuclei.

OTR-IR neurons were observed evidently from the rostral to the caudal portions of the NTS either in the RWIS rats or the unstressed rats (Figure 6). Compared with the DMV, in the NTS, OTR-IR neurons, mainly located in the mNTS, imNTS and vNTS, were fusiform or round profiles without any neuronal processes, and smaller in size with a scanty cytoplasm that was hardly distinguished in double immunostained neurons. The major location of Fos+OTR-IR neurons was the intermediate part of the NTS, and less of them was present in its posterior and anterior portions, but the difference was't significant (*F*_2,12_ = 0.336, *P* = 0.721). Within the NTS, most of Fos+OTR-IR neurons were confined to the imNTS and vNTS. Overall, in the NTS, Fos+OTR-IR neurons represented in 9.79%±0.96% of total OTR-IR neurons in the RWIS group, and compared with 5.25%±0.09% in the control group, the difference was significant (*P*<0.01).

V_1b_R-IR neurons located evidently from the rostral to the caudal portions of the NTS either in the RWIS rats or the unstressed rats (Figure 7). The profile and distribution of V_1b_R-IR neurons were similar with OTR-IR neurons. The location of Fos+V_1b_R-IR neurons was even within the different portions of the NTS (*F*_2,12_ = 0.059, *P* = 0.943). Within the NTS, most of Fos+V_1b_R-IR neurons were confined to the imNTS and dlNTS. Overall, in the NTS, Fos+V_1b_R-IR neurons represented in 8.16%±0.82% of total V_1b_R-IR neurons in the RWIS group, and compared with that in the control group (3.57%±0.38%), the difference was significant (*P*<0.01). Furthermore, there was significant difference in the percentage of Fos+V_1b_R-IR neurons of the Fos-IR nuclei between the RWIS group (49.43%±3.36%) and the control group (27.03±2.91%) (*P*<0.01).

## Discussion

In the present study, RWIS for 1 h evoked a marked neuronal activation in the SON, PVN, DMV and NTS, a pattern consistent with that reported previously by us under similar conditions [3]. The characterizations of these activated neurons in the PVN, SON, DMV and NTS were assessed by bouble-staining. Morphological aspects and chemical coding revealed that the RWIS activates the hypothalamic oxytocinergic and vasopressinergic neurons and these neurons may project to the NTS and DMV mediated by OTR and V_1b_R.

### Oxytocinergic and Vasopressinergic hypothalamic neurons involved in the mediation of signals induced by RWIS

PVN and SON, as the main nuclei of the anterior hypothalamus, not only innervate areas of the brain known to be involved in cardiovascular regulation, but might be stimulus-dependent a variety of stimuli [19]-[26], [39]-[40]. In the present study, the marked activation of neurons induced by RWIS encompasses mainly the SON and PVN, which accounts for the important role of the anterior hypothalamus in response to the RWIS. Furthermore, the pattern emerging from the results of immunohistochemistry revealed a topographically distinct distribution of activated neurons in the subdivisions of the PVN and in the SON. In the SON Fos-IR nuclei were evenly distributed, while in the PVN, the PaMP, PaLM and PaMM subdivisions showed a robust increase in Fos-IR nuclei, the PaAP, PaDC and PaV subdivisions displayed a modest Fos expression, and only a few of Fos-IR nuclei scattered in the Pe and PaPo. This suggests that different subdivisions of the PVN may take a different role in this response and the PVN may be involved in the regulation of a variety of central neural functions.

Swanson LW and Sawchenko PE reported that OT-containing neurons are massed in the central core of the posterior magnocellular subdivision of the PVN and the dosolateral part of the SON, while AVP are massed in the circumference of the PVN and the ventromedial portion of the SON [41]. In the present study, OT-IR and AVP-IR neurons mostly located in the medial portion of the PVN, including PaMM and PaLM, but in the SON the OT-IR neurons mainly located in the dorsal part, while AVP-IR evenly distributed within the SON. The distribution of the OT- and AVP-IR perikarya in the PVN and SON co-responded well with the principal distribution of OT- and AVP-IR neurons reported in other rats or mice studies [25]–[27]. Double labeling showed that the majority of oxytocinergic and vasopressinergic neurons in the hypothalamus, including the PVN and SON, expressed Fos, which indicated that these neurons were directly or indirectly activated by the RWIS. In the PVN, Fos-IR nuclei were indentified in 31% of OT-IR and 40% of AVP-IR neurons, while in the SON it represented 28% of OT-IR and 53% of AVP-IR neurons. These findings indicate that PVN oxytocinergic and vasopressinergic neurons are all prominently activated by the RWIS, and vasopressinergic neurons in the SON are key component in the integration of stress-related signals, while oxytocinergic neurons seem to participated to a less extent. The different OT and AVP ratio in the neuroendocrine activation of the PVN and SON expends previous studies showing a differential activation of oxytocinergic and AVP-IR neurons in response to a variety of stimuli. For example, proximal colon distension induced Fos expression in about 81% of OT-containing neurons and 18% of vasopressin neurons in the PVN, while in the SON 36% and 16%, respectively [27]. Osmotic stress activated 38%-45% of the oxytocinergic and 62%-67% of the vasopressinergic neurons in the PVN, and more than 50% of oxytocinergic and vasopressinergic neurons in the SON, while immobilization stress induced Fos expression in only 4%-8% of the oxytocinergic neurons and 10%-15% of the vasopressinergic neurons in the PVN, and less than 4% of oxytocinergic and vasopressinergic neurons in the SON [26]. These observations and the present results suggest that activations of oxytocinergic and vasopressinergic neurons in the PVN and SON depends on the style of stressors and that the hypothalamic oxytocinergic and vasopressinergic neurons may be involved in the regulation of a variety of central neural functions.

### Oxytocin-sensitive and Vasopressin-sensitive neurons in the medullary visceral zone involved in the mediation of signals induced by RWIS

The NTS is the major recipient of visceral afferent information arising from various regions of the gastrointestinal tract, while the DMV is a visceral motor nucleus, and morphometry showed that the vagal parasympathetic neurons innervating the stomach are largely located in the DMV [42]–[44]. RWIS for 1 h induced a robust increase in Fos-IR nuclei in the NTS and DMV, which indicated that the NTS and DMV neurons were responsive to the RWIS. These results are in good agreement with our previous reports that RWIS induced the expression of Fos protein in the NTS and DMV [3]–[4]. The difference of the activated neurons in different portions of the NTS and DMV may be due to their different roles in modulating the gastric functions during RWIS [3], [6]–[7], [45]–[46].

Previous studies found that OT and AVP neurons in the PVN project to the NTS, DMV and spinal cord at the level of sympathetic preganglionic neurons, where OT and V1 receptors are present [11], [28], [47]. In the present study, more than 10% of OTR-IR and V_1b_R-IR neurons in the DMV were activated in the RWIS rats while less than 3% in the unstressed rats. In the NTS, the percentages of Fos-IR neurons in the OTR-IR and V_1b_R-IR neurons were 10% and 8%, while 5% and 3% in the unstressed rats, respectively. The significant difference of the ratio between the RWIS and unstressed groups indicates that the OT-sensitive and AVP-sensitive neurons may be involved in the modulation of the gastric dysfunction during the RWIS. But, there was no difference in the number of Fos+OTR or V_1b_R double-labeled neurons in any portions of the DMV and NTS. This result may suggest that OT or AVP-containing neurons are involved in the regulation of a variety of central neural functions. Oxytocinergic and vasopressinergic neurons in the PVN and SON receive afferent projections from visceral centers, and then release AVP or OT, which have been shown to stimulate autonomic neurons in the NTS and DMV, thereby improving vagal outflow and augmenting reflex [48]–[50]. Microinjection of OT into the rat DVC activates gastric projecting neurons in the NTS and DMV [18], and inhibits gastric motility [51]. Moreover, the decrease in gastric motility in response to PVN stimulation is blocked by microinjection of OT receptor antagonists in the DMV [51].

### Oxytocinergic and vasopressinergic system may activate the parasympathetic outflows to modulate the signals induced by RWIS

It is well known that the activation of hypothalamic-pituitary-adrenocortical (HPA) axis by stress is an important regulatory mechanism used by most mammals to maintain homeostasis after multiple types of challenges. Whether the activation of OT- and AVP-containing neurons in the PVN and SON in response to the RWIS is dependent on the activation of HPA axis [52]? In the present study, the founding of numerous of Fos-IR nuclei in the PaMP, where corticotropin-releasing hormone (CRF) neurons are messed, seemed to be agreement with this viewpoint.

But our previous study found that in the zona fasciculata of the adrenal cortex, where glucocorticoid was synthesized, no Fos expression was observed during the RWIS. Neuroanatomic studies have shown that hypothalamic OT- and AVP-containing neurons receive neural projections from the NTS and, in turn, send projections to visceral centers in the medullary visceral zone, including the NTS, DMV and spinal cord [53]. Furthermore, bilateral vagotomy attenuated the effect of electric stimulation of the PVN on stress ulcers [23]. In addition, in the present study, numerous of Fos+OTR-IR and Fos+V_1b_R-IR double labeled neurons were observed in the DMV and NTS. These results support and expend the viewpoint that PVN neurons receive inputs from visceral receptors and then release AVP or OT, part of which project to the NTS or DMV and then activate the parasympathetic outflows [50].

In summary, RWIS for 1 h results in the activations of a large population of OT- and AVP- containing neurons, 31% and 40% in the PVN, 28% and 53% in the SON. In addition, RWIS activates more than 10% of OT and AVP sensitive neurons in the DMV, while lower ratio in the NTS. Thus it is hypothesized that OT- and AVP-containing neurons in the PVN and SON, activated by RWIS, would project to the NTS or DMV, mediated by OTR and V_1b_R, and then the DMV in turn provide the preganglionic efferent fibers to regulate gastric information. But, up to now, no report has been found to describe that the highest density of OT- and AVP-immunoreactive fibers and the terminals in the DMV and NTS originated in the PVN only or both the PVN and SON. Thus, mechanism of the oxytocinergic and vasopressinergic system during the RWIS needs to be further investigated.
